# Is there evidence behind pre- or perioperative cognitive training in gynaecological patients on the prevention of perioperative cognitive dysfunction? A review

**DOI:** 10.1007/s00404-021-06315-0

**Published:** 2021-12-07

**Authors:** Sophia Volz, Franziska Koch, Davud Dayan, Miriam Upadhyay, Stephanie Otto, Fabienne Schochter, Wolfgang Janni, Florian Ebner

**Affiliations:** 1 Frauenklinik, Universität Ulm, Prittwitzstr, Ulm, Germany; 2 Helios Klinikum Schwerin, Chirurgie, Schwerin, Germany; 3 Helios Amper Klinikum Dachau, Krankenhausstr. 15, 85221 Dachau, Germany; 4 Ulm University Hospital, Comprehensive Cancer Center Ulm (CCCU), Ulm, Germany

**Keywords:** Cognitive, Prehabilitation, Surgery, ERAS, Recovery, Prevention

## Abstract

**Purpose:**

Perioperative cognitive dysfunction can be observed in all age groups of patients. Sometimes, this is more stressful to the patient than the actual surgical wound. Enhanced recovery after surgery pathways screen for patients at risk and lead to early post-surgical intervention. To prevent cognitive dysfunction, a prehabilitation approach might be useful.

**Methods:**

This systematic literature review provides an overview on the current knowledge on prehabilitation for cognitive dysfunction for gynaecological patients by searching the National Library of Medicine (PubMed) in February 2020 to identify publications regarding presurgical cognitive training with three different search terms.

**Results:**

501 articles were identified and after screening for eligibility five were left for further analysis. Generally, cognitive function is split into several cognitive aspects like anxiety or memory, speed, attention, flexibility or problem-solving functions. Each of these aspects can/need to be trained to show an improvement after general anaesthesia. Training possibilities range from relaxation methods via music, one-on-one personal training sessions to electronically supported training units.

**Conclusion:**

Prehabilitation of the cognitive function can be split in different cognitive domains. Each of these domains seem to be influenced by training. The training itself can be based on applications or known relaxation methods or even old-fashioned board games. The evidence is, however, still low and there is a need for further studies.

## Introduction

Enhanced recovery pathways after surgery (ERAS) are evolving in gynaecological surgery [1, 2]. The ERAS pathway defines guidelines from before admission to after discharge to reduce the stress response, optimise physiological function, and facilitate recovery. Exercises in the time period prior to admission are also known as prehabilitation. ERAS pathways rarely support the cognitive side of the recovery. The aim of this review is an overview of existing evidence regarding gynaecological patient-based cognitive training prior to surgery.

Other disciplines have published research on perioperative neurocognitive dysfunction [3, 4]⁠, investigating biomarkers, anaesthetic techniques and post-surgical training [5]⁠. There are several aspects of cognitive function that might influence and could possibly be improved by the patients themselves. Anxiety has been known to impact all aspects of patient care. Further cognitive aspects are memory, speed, attention, flexibility, and problem-solving functions. With patients themselves having different abilities in these aspects, it is difficult to prove a valid cognitive training in a prospective randomised study. The following article provides an overview of the current literature and its possible clinical implementation with a focus on the ‘do it yourself’ patient.

## Materials and methods

**Fig. 1:**
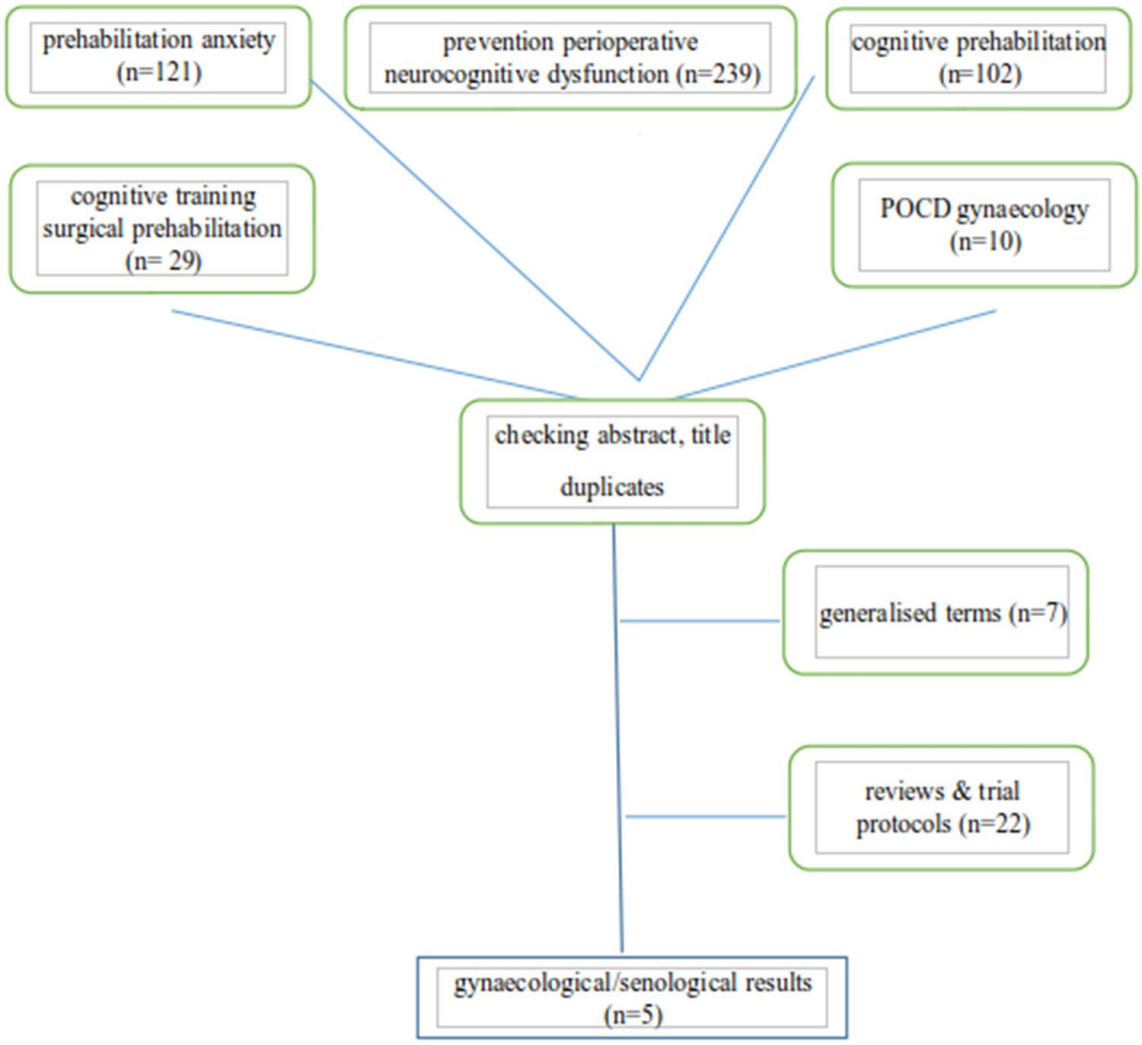
Flowchart of search results

The National Library of Medicine (PubMed) was searched without filters for time or language in February 2020 to identify publications regarding presurgical cognitive training. This search was updated during the review process in August 2021 (Time filter: start 31/1/2020; end date 31/7/2021). The title and abstract of the results were screened for relevance by two researchers (FE, FK) looking for cognitive prehabilitation in gynaecological surgeries. The remaining articles, if applicable references, and suggested relevant similar topic articles were included in the results. The search terms were (I) ‘prehabilitation anxiety ‘(*n* = 121)’, cognitive prehabilitation ‘(*n* = 102)’, cognitive surgical ‘(*n* = 24,794)’ and adding (II) cognitive training surgical prehabilitation ‘reduced the number of results (*n* = 29)’. Additionally (III) prevention perioperative neurocognitive dysfunction and (IV) ‘POCD gynaecology’ were searched and resulted in 239 and 10 hits (Fig. 1).

## Results

After screening the title and abstract for relevance, five results met the strict inclusion criteria (gynaecological/breast surgery/cognitive prehabilitation [6–10]⁠⁠ of the search. The gynaecological diagnoses found were breast cancer [9, 11]⁠, caesarean section [7]⁠, endometrium cancer [10]⁠ and cervical cancer [8]⁠. The number of included patients ranged from 0 [8]⁠ to 75 [11]⁠ and the type of publications was randomised trials [9, 11, 12]⁠, case reports [10]⁠ and a protocol for a systematic review [8]⁠.

Several abstracts (*n* = 7) used generalised terms like ‘surgical patients’ or ‘cytoreductive surgery’ and were additionally included for further information. Prehabilitation reviews and trial protocols were screened for relevant references and insights. Further details on the literature found are provided in Table 1.

**Table 1:** Literature details

Results
Prevention of postoperative cognitive dysfunction is the best treatment
Multimodal prehabilitation is a feasible intervention
Significantly reduced the anxiety
Short-term benefit
Further studies should be encouraged
Preoperative prehabilitation programme may decrease the risk of postoperative cognitive dysfunction and improve functional recovery
N/a
No anxiety difference, but more exercises
Intervention better than control
Screening is necessary
No difference, technical difficulties, commitment issues
Intervention lowered delirium risk in patients who were at least minimally compliant
SCD occurred after anesthesia and surgery
N/a

### Evaluated parameters

The reduction of anxiety is a main concern for any medical intervention. In our results, various tests were used covering more aspects of cognitive function. The interventions aimed at improving these results. Anxiety was measured explicitly with the Spielberger’s State Trait Anxiety Inventory (STAI) in Che et al. Garssen and Ertug (Turkish version) [7, 9, 13]⁠. Other presurgical tests were the Short Form-36 Health-Related Quality of Life Measure (SF-36) (also [14, 15]⁠) and Repeatable Battery for the Assessment of Neuropsychological Status (RBANS™) three times up to 8 weeks after surgery[10]⁠. Salivatory cortison levels were measured three times a day up to the second day after surgery along the STAI. and Garssen with several questionnaires to measure pain [9, 13]⁠, quality of life, sleeping problems, the subscale from the Profile of Mood States for the depression and fatigue as well as questionnaires designed by the Garssen et al. The questionnaires were handed out at different times before and after surgery.

Wu et al. used three questionnaires for patient-reported outcomes [Short Form-12 Health-Related Quality of Life Measure (SF-12), Hospital Anxiety and Depression Scale (HADS) and Shoulder Pain and Disability Index (SPADI)] at baseline and 6 weeks post-surgery [11]⁠. Vlisides et al. used a 3-min diagnostic assessment [16]⁠ (3D-Confusion Assessment Method, CAM) to screen for delirium and the NIH Toolbox Cognition Battery (NIH-TB) for further cognitive evaluation. The NIH Toolbox Cognition Battery consists of tests of multiple constructs and includes seven tests covering 6 cognitive abilities.

In the Neurobics Trial, brief Confusion Assessment Method, Memorial Delirium Assessment Scale were used as evaluation parameters alongside the Mini-Mental Status Exam (MMSE), Self-Administered Gerocognitive Form (SAGE), Geriatric Depression Scale, Charlson Comorbidity Index, and Postoperative Quality Recovery Scale (PQRS) [14]. Though focusing on physical fitness, the randomised feasibility trial of Steffens et al. also recorded a Depression, Anxiety, Stress Scale (DASS) score and a visual analogue scale (VAS) score.

The earliest report on elective surgical patients regarding the effect of preoperative instructions found was published in 1987. The authors developed an exercise checklist and also used the STAI [17]⁠.

### Prehabilitation start

The start of the interventions ranged from 6 weeks prior to the day of the surgery. This final last minute intervention was done by Ertug et al. for relaxation prior to theatre [13]⁠. Che et al. [7] started with the intervention the evening before the elective surgery⁠. In the breast cancer trials 6 days [9]⁠ and 2 weeks [11]⁠ prior to surgery were used. Mikulaninec started, depending on the study arm, 1 week prior to admission or postadmission with the education [17]⁠. The participants were encouraged to train at least seven times prior to surgery, ideally on a daily base for 20 min in the Vlisides study [16]⁠. The aim of the Neurobics trial was to have 10 h of tablet-based training with at least 8 days prior to surgery [14], thus not looking at the time to surgery but at the time spent in active homebased training. In the case report of Carli et al., the prehabilitation started the programme three weeks before the surgery [10]⁠. Steffens et al. [15]⁠ started 2–6 weeks prior to the scheduled surgery.

### Cognitive prehabilitation programme

The term prehabilitation is used for various training aspects prior to an intervention. Coming from ERAS, the physiological and psychological aspects need considering. Multimodal training programmes consist usually of physical, dietary and cognitive training units. There are various aspects like anxiety, fatigue and stress that are usually addressed in the cognitive training. Wu et al. [11] identified patients with raised anxiety and/or depression with the before-mentioned screening questionnaires⁠. Patients were then assigned to a qualified counsellor and had at least one session. Here, mindfulness techniques were taught. This included breathing techniques, mental training and meditation.

Garssen et al. [9] focused entirely on stress management training (SMT). In their prospective study, patients were randomly assigned to a SMT intervention or no intervention. The SMT consisted of four sessions with a psychologist. Here, relaxation, guided imagery techniques and counselling helped with active coping, alert relaxation and supported a positive attitude. Additionally, an audio CD with instructions was handed to the patients for home exercise.

Carli et al.’s [10] patient had lower scores on the SF-36 confirming the psychologists reports with depressive symptoms. No special cognitive training was scheduled. This was also the case for Steffens et al. ‘s trial [15]. Both trails had only individualised physical training sessions for the participants.

Che et al. [7] showed the trial group an 8.5-min informational video on the application of anaesthesia for an elective caesarean section, Mikulaninec [17] posted out a 14-page educational booklet and in Ertug’s study, patients listened to nature sounds for relaxation [13]. The internet-based cognitive training battery from Vlisides specifically targeted the executive function, attention, working memory and visuospatial processing in a gaming approach [16]. The Neurobics trial used a mobile app to train the patients at least 1 h a day [14]. The training programme was adjusted to the individual results of the tests.

### Outcome

The trails without cognitive training [10, 15]⁠ still resulted in a cognitive change. It was noted that the 88-year-old patient with an improved 6-min walk test also improved the physical and mental components of the SF-36 [10]⁠. This was attributed to the social support. Despite the low participant numbers, the mental part of the SF-36 showed less amplitude in the trial group compared to the control group [15]⁠. Also the pain score remained below the control group in the trail group. However, no statistical significant difference was found.

The outcome of the video interventions showed no difference in the salivary cortisone levels between the two groups. The anxiety levels were significantly lower in the video intervention group proving the hypothesis that informational videos can significantly reduce maternal anxiety and increase the maternal satisfaction [7]⁠. However, it failed to show a benefit for postoperative recovery. Mikulaninec had similar results. Here, the knowledge transfer was significantly different in group teaching and more educated patients, but no statistical difference was found in anxiety levels [17]⁠. Wu et al. [11] reported a significant difference in the HADS anxiety scores but no difference in the SF-12 mental or HADS depression scores⁠.

Significant results were published by Garssen et al. [9]⁠ for various parameters like quality of life (day before and 2 days after surgery, 3 months after), fatigue (days 2 and 5 and 3 months after surgery) and depression (day 5). Further beneficial correlations were found with more frequent users of the CD and fatigue and sleep problems at 90 days post-surgery. The nature sound/relaxation from Ertug et al. [13]⁠ showed significant reduction in VAS and STAI scores after the intervention and 30 min after, compared to the control group. No difference was found in between the two intervention groups. This was similar in the study from Vlisides et al. Here no statistically significant difference was found [16]⁠. In the post hoc explorative correlations analysis, trends were seen for patients who trained more and started with a higher pre-training baseline score. The same was found in the Neurobics trial [14]⁠. Here, the initial results were not significantly different, but once the study group excluded patients who had not used the training, a significant difference was found between the study and the intervention group regarding postoperative delirium.

## Discussion

Enhanced recovery after surgery includes the physiological and psychological aspects of the patients. The post-surgical cognitive impairment is known (33,071,859) and influences the recovery [5]⁠. Current standard of treatment is the early training after surgery with studies investigating the training towards surgery in prehabilitation concepts. Screening tools to identify patients at risk and early post-surgical interventions are published [18]⁠ and have been reviewed and clinically implemented [19]⁠. The understanding of the basic pathomechanisms is complex and is just beginning to understand. The term cognitive impairment implicates any impairment in any cognitive domains. But each domain needs training with different stimuli. Anxiety for example can easily be reduced with information and personal conversations [7, 11, 17]⁠. It helps if hospital staff are empathic and spend time with the patient answering questions and helping with concerns [7, 13]⁠. On the other hand, is it possible the patient him-/herself can train relaxation techniques like breathing, meditation or listen to relaxing sounds to calm down prior to surgery, all of which have shown positive effects in trial by improving the used screening scores [7, 9, 13, 17]⁠.

Further cognitive domains like memory, attention or problem-solving were in the focus of Vlisides and the Neurobics trial with significant benefits after excluding non-compliant participants [14, 16]⁠. With automatised electronic training possibilities, this might become an option in the future for effective individualised cognitive training [14, 20]⁠. Electronic training enables the researchers to monitor the actual time spent training and the results, thus improving the data quality. But the optimal training mode still needs to be determined and as other authors report there is a substantial number of patients hesitating to train online Vlisides et al. (2019), Wu et al., O’Gara et al. The editorial of Culley et al. [21]⁠ for example comments on Kawano et al. [22]⁠who demonstrated a positive influence in rats with cognitive stimuli. Culley et al. concluded that ‘…Finally, cognitive training might be unrealistic preoperatively because seniors are typically not facile with technology…’ but the results of Kawano are praised for the novelty and the perspective on exercise, as medicine, is an exciting prospect.

Also, there are easy non-digital alternatives to train the patients’ cognitive abilities [23]⁠. Here, a regular set of playing cards will be used as a memory game in the future trial. Butz et al. [24] have posted a study protocol using paper and pencil for cognitive training in cardiac surgery⁠. Or Estrada-Plana et al. [25] used various modern commercially available games in two randomised trials⁠. They showed a significant improvement in semantic verbal fluency and conclude that ‘Modern board and card games could be an effective cognitive intervention…’. And in the case report of Carli, the social support contributed to the cognitive improvement [10]⁠. So, it could be expected that even classic games like bridge or Ludo may benefit the patients’ cognitive function prior to surgery but this is still to be proven.

To relax or reduce anxiety, there are promising results from listening to nature sounds or relaxation [13]⁠. Other methods like Mindfulness-Based Stress Reduction (MBSR) and yoga [26–28]⁠ have shown to influence stress hormones and pro-inflammatory cytokines [29–31]⁠. However, these benefits have not (yet) been published in the gynaecological–surgical context in randomised trials.

## Conclusion

There is an increasing number of cognitive prehabilitation trials indicating benefits for the patients. Trials with specified procedures are published for breast cancer surgery and caesarean section but not for most other gynaecologic surgeries. To monitor possible effects, several screening tools are available but a standard tool is still to be defined.

Physical and mental training seems to be interconnected and a combined training should be beneficial for compliant patients. The best evidence is available for a reduction of presurgical anxiety. The mode of sharing information in terms of booklet, video or audio seems to be irrelevant. By implementing newer technologies, further cognitive domains can be stimulated. It seems that prehabilitation of the cognitive function improves at least for these trained domains (anxiety, memory, etc.). The combination of cognitive and physical training and dietary optimisation may improve the overall cognitive outcome of the patients and their quality of life in general.

## Data Availability

Not applicable.
